# An information-theoretic evaluation framework for CNN–LSTM-based Alzheimer’s disease classification from structural MRI

**DOI:** 10.1038/s41598-026-56939-y

**Published:** 2026-06-09

**Authors:** Shiva Sanati, Elias Rahimi, Ghosheh Abed Hodtani, Saeid Eslami

**Affiliations:** 1 Department of Medical Informatics, Faculty of Medicine, Mashhad University of Medical Sciences, Mashhad, Iran; 2 Department of Computer Engineering, Bahar Institute of Higher Education, Mashhad, Iran; 3 Departments of Electrical Engineering and AI, Ferdowsi University of Mashhad, Mashhad, Iran; 4 Department of Medical Informatics, University of Amsterdam, Amsterdam, NLD Netherlands

**Keywords:** Alzheimer’s disease, Structural MRI, CNN–LSTM, Post-hoc information-theoretic evaluation, Renyi divergence, GAN-based augmentation, Computational biology and bioinformatics, Diseases, Mathematics and computing, Neurology, Neuroscience

## Abstract

Early detection of Alzheimer’s disease (AD) is important because of its progressive impact on cognitive function. This study presents a CNN–LSTM-based framework for three-class AD classification from structural MRI, with the primary contribution being a post-hoc information-theoretic evaluation strategy rather than a new network architecture. Experiments were conducted using 827 ADNI subjects, including normal controls (NC: 340), mild cognitive impairment (MCI: 307), and AD (180). To mitigate data scarcity and improve training diversity, GAN-based augmentation was applied only to the training data, while validation and test subjects were kept separate. In addition to conventional metrics, trained models were evaluated using Renyi mutual information, Renyi divergence, and Henze–Penrose divergence to quantify information preservation, representation stability, and distributional alignment. Under a subject-level evaluation protocol, the CNN–LSTM model achieved 96.7% accuracy and outperformed evaluated benchmark architectures under the same protocol. The information-theoretic measures provided complementary evidence for comparing model behavior beyond accuracy, particularly regarding information retention and output-distribution alignment. Overall, the findings suggest that post-hoc information-theoretic analysis can support more transparent assessment of MRI-based AD classification models. However, external validation on independent multi-center datasets is required before clinical deployment can be considered.

## Introduction

Alzheimer’s disease (AD) is a progressive neurodegenerative disorder and the leading cause of dementia among the elderly, affecting memory, reasoning, and behavior. AD profoundly affects cognitive function, creating substantial challenges for patients, their loved ones, and healthcare infrastructures worldwide. Given its prevalence and impact, early and accurate diagnosis is imperative to facilitate timely interventions and potentially alter the disease trajectory. With global dementia cases projected to reach 139 million by 2050, early detection strategies are more crucial than ever^1^.

Recent advances in medical imaging—especially magnetic resonance imaging (MRI)—combined with deep learning, have enabled non-invasive methods for identifying AD in its initial stages. MRI offers high-resolution structural insights into the brain, making it invaluable for detecting changes in vulnerable regions such as the hippocampus, thalamus, medial temporal lobe, cingulate gyrus, parietal cortex, and superior temporal gyrus^2^.

Among these, hippocampal atrophy is widely acknowledged as a hallmark of early AD and a key biomarker for differential diagnosis. Individuals presenting with early cognitive deficits but not meeting the criteria for AD are often diagnosed with Mild Cognitive Impairment (MCI), a transitional state. Critically, around 20% of MCI cases progress to AD each year^2^, further underscoring the urgency of precise diagnostic approaches.

**Fig. 1:**
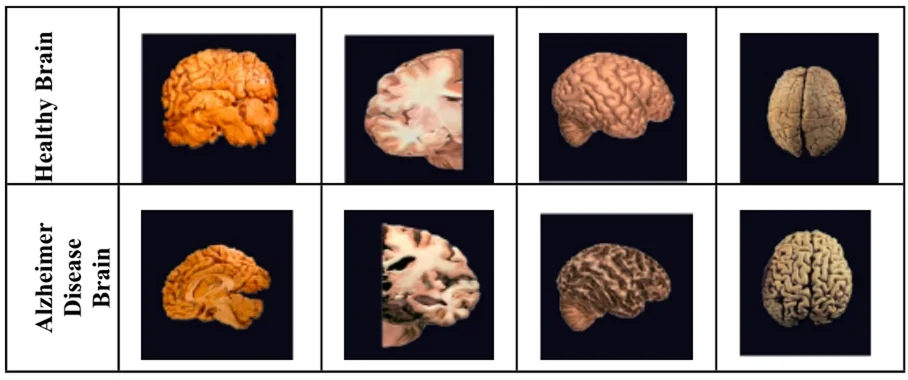
Representative structural comparison between a cognitively normal brain and a brain affected by Alzheimer’s disease, illustrating MRI-detectable patterns commonly associated with AD, including cortical atrophy, ventricular enlargement, and reduced brain volume.

Figure 1 provides a visual comparison between a healthy brain and one affected by Alzheimer’s disease, clearly illustrating key pathological features such as cortical atrophy and ventricular enlargement. These structural abnormalities are typically quantified using voxel-based morphometry (VBM) techniques, often implemented through the SPM toolbox applied to MRI scans^3^.

Traditional diagnostic methods often grapple with the intricate, high-dimensional nature of MRI data. In contrast, deep learning approaches, such as Convolutional Neural Networks (CNNs) and Recurrent neural networks, can capture spatial patterns and ordered anatomical relationships within neuroimaging data^4,5^. In the present study, the LSTM component is used to process anatomically ordered MRI slice representations rather than to model true temporal evolution.

Notably, Han et al.^6^ developed a multi-template network with meta-information regularization to improve Alzheimer’s diagnosis using structural MRI scans, achieving robust feature learning for AD classification. Similarly, Heravi et al.^7^ proposed a correntropy-based backpropagation algorithm to enhance neural network training robustness in noisy conditions, providing insights for optimizing architectures like those used in neuroimaging.

In this study, we employ a hybrid CNN–LSTM framework that combines CNN-based intra-slice spatial feature extraction with LSTM-based modeling of ordered inter-slice dependencies for classifying MRI scans into three diagnostic categories: AD, MCI, and Normal Controls (NC). CNNs detect structural signatures such as hippocampal shrinkage and cortical thinning, while LSTMs are employed to model structured inter-slice dependencies and spatial continuity across ordered MRI slices, thereby enabling integrated modeling of structural patterns across MRI representations.

MRI data, whether processed as 2D slices or 3D volumes, holds critical structural cues. Voxel-based morphometry (VBM) is one of the most reliable methods for quantifying brain atrophy using MRI, allowing researchers to compare gray matter density across different populations^3,8,9^. Several studies have demonstrated the effectiveness of VBM in identifying AD-related changes, especially in hippocampal and cortical structures^10–12^.

In this work, we adopt preprocessing strategies using the Statistical Parametric Mapping (SPM) toolbox and the CAT12 framework, segmenting brain structures and aligning them in standard MNI space. Prior research by Sanati et al. has shown the efficacy of this pipeline in both segmentation and volumetric analysis^13–16^. Moreover, to address data scarcity and enhance generalizability, Generative Adversarial Networks (GANs) are employed to synthesize high-fidelity MRI samples, thereby augmenting the dataset and improving classification robustness.

Although several deep learning architectures have shown promising performance in MRI-based Alzheimer’s disease classification—including Siamese CNNs, 3D CNNs, transformed-domain CNNs, and ensemble-based approaches^17–20^, most studies still rely primarily on conventional outcome-based metrics such as accuracy, sensitivity, specificity, precision, and F1-score. These metrics are essential for evaluating predictive performance, but they provide limited insight into how learned representations preserve diagnostically relevant information, how well output distributions remain aligned with class structure, or how different model configurations affect representation stability.

Information-theoretic analysis has previously been used to study representation learning, information preservation, and learning behavior in neural systems^21,22^. Building on this motivation, the present study introduces an information-theoretic evaluation framework for assessing CNN–LSTM-based Alzheimer’s disease classification from structural MRI. The purpose of this framework is not to propose a new CNN–LSTM architecture or an information-theoretic training objective. Instead, Renyi mutual information, Renyi divergence, and Henze–Penrose divergence are applied as post-hoc analytical measures to complement conventional classification metrics. These measures are used to evaluate information preservation, class separability, and distributional alignment across model configurations and benchmark architectures.

The CNN–LSTM model used in this study follows a standard hybrid design in which convolutional layers extract intra-slice spatial features and LSTM layers model ordered inter-slice spatial dependencies. The methodological contribution of this work lies in the systematic evaluation of such learned representations through information-theoretic criteria, rather than in claiming architectural novelty. This distinction is important because the proposed measures are intended to support more transparent model comparison, hyperparameter interpretation, and reliability assessment in neuroimaging studies.

The main contributions of this study are summarized as follows:

1. We present a post-hoc information-theoretic evaluation framework for CNN–LSTM-based Alzheimer’s disease classification from structural MRI.
2. We apply Renyi mutual information, Renyi divergence, and Henze–Penrose divergence to assess information preservation, representation stability, and distributional alignment beyond conventional accuracy-based metrics.
3. We compare the CNN–LSTM framework with benchmark architectures under a consistent evaluation protocol, including both conventional classification metrics and information-theoretic measures.
4. We evaluate the proposed framework under subject-level data splitting to reduce leakage risk and to provide a more reliable assessment of model performance on ADNI structural MRI data.

The remainder of this paper is organized as follows. Section “Related work” reviews related work and background concepts. Section “Proposed method” presents the proposed methodology in detail. Section “Proposed method evaluation and results” reports the experimental setup and quantitative results. Section “Discussion” discusses the findings and limitations. Finally, section “Conclusion” concludes the paper.

## Related work

This section briefly introduces the core components of the proposed CNN-LSTM hybrid model, focusing on their relevance to Alzheimer’s disease (AD) classification using MRI data. We outline the roles of Convolutional Neural Networks (CNNs), Long Short-Term Memory (LSTM) networks, and information-theoretic measures to provide context for the proposed evaluation framework.

### Convolutional neural networks

Convolutional Neural Networks (CNNs) are powerful tools for analyzing complex neuroimaging data, such as MRI scans, due to their ability to extract hierarchical spatial features with minimal preprocessing^23,24^.

In Alzheimer’s disease (AD) detection, CNNs are particularly effective at identifying structural biomarkers, including hippocampal atrophy, cortical thinning, and ventricular enlargement, which are critical for distinguishing AD, MCI, and NC^5,12^.

In our proposed CNN-LSTM hybrid framework, CNNs act as a robust encoder, capturing intricate spatial patterns from preprocessed gray matter MRI images segmented using the CAT12 and SPM12 toolboxes. Unlike standalone CNN models.

widely applied to AD classification^4,17,25–27^, our approach integrates CNNs with Long Short-Term Memory (LSTM) layers to exploit both spatial and ordered inter-slice spatial dependencies across MRI slices. For instance, Payan and Montana^4^ leveraged 3D CNNs to preserve volumetric information, while Choi and Lee^26^ combined multiple deep networks through an ensemble generalization strategy for MRI-based AD classification. Sarraf and Tofighi^27^ employed CNNs for AD differentiation, and Manzak et al.^25^ combined CNNs with Random Forests for enhanced classification. Building on these advancements, the CNN component in the present framework comprises four convolutional layers with 64, 128, 256, and 512 filters to extract AD-relevant spatial features from preprocessed MRI slices. When combined with LSTM-based modeling of ordered inter-slice spatial dependencies, the framework achieved a mean subject-level cross-validation accuracy of 96.7% and showed higher performance than evaluated benchmark models such as ResNet-50 and VGG16 under the same experimental protocol (section “Comparative evaluation with state-of-the-art models”).

Recent deep learning studies in medical image analysis have increasingly emphasized attention mechanisms, feature fusion, semi-supervised learning, transformer-based representation learning, and foundation-model-inspired approaches. For example, SSMD-UNet used a semi-supervised multi-task decoder strategy for diabetic retinopathy lesion segmentation^28^, while attention-augmented CNN studies showed that SE and CBAM modules can improve feature recalibration and generalization across medical image classification tasks^29^. Hierarchical feature-fusion and ensemble strategies have also been explored for brain MRI classification^30^, and hybrid CNN–transformer models have been used to combine local convolutional features with global dependency modeling^31^. More recently, regularized transformer models with adaptive token fusion have been proposed for Alzheimer’s disease diagnosis from brain MRI by improving the integration of local anatomical features and global contextual information^32^. SAM-inspired frameworks combined with hierarchical or enhanced attention mechanisms have also been investigated for Alzheimer’s disease detection, reflecting the growing interest in segmentation-aware representations and attention-based feature refinement in neuroimaging^33^. Beyond Alzheimer’s disease, large-filter and report-guided alignment networks for chest X-ray diagnosis illustrate how task-specific visual feature extraction and auxiliary textual or clinical information can improve medical image classification^34^. These studies mainly advance architecture design, multimodal representation learning, and attention-based feature refinement. In contrast, the present work focuses on post-hoc information-theoretic evaluation of CNN–LSTM representations for MRI-based Alzheimer’s disease classification rather than proposing a transformer-, SAM-, or report-guided architecture.

### Long short term memory (LSTM)

Long Short-Term Memory (LSTM) networks are recurrent neural network variants designed to model dependencies in ordered data. In this study, LSTM layers are not used to model temporal progression, because structural MRI slices do not represent time-dependent observations. Instead, the LSTM component is applied to anatomically ordered 2D MRI slices extracted from 3D volumes, with the goal of capturing inter-slice spatial dependencies and continuity across adjacent brain regions.

This design allows CNN-extracted intra-slice features to be further analyzed in relation to neighboring slices, which may help preserve spatial context that would be ignored by a purely slice-independent 2D CNN. However, this approach should be interpreted as a computationally efficient sequence-based approximation of volumetric structure rather than a substitute for full 3D modeling. Full 3D CNNs and transformer-based volumetric attention models may capture richer spatial relationships, but they generally require greater computational resources and larger training datasets. Therefore, the CNN–LSTM configuration used in this study represents a compromise between computational feasibility and the need to model inter-slice anatomical continuity.

### Information-theoretic measures for model evaluation

Deep learning models have achieved remarkable success across diverse fields such as image classification, natural language processing, and medical diagnostics. However, despite these advances, understanding the internal mechanisms that drive model performance remains a significant challenge. Information theory offers a powerful mathematical framework for analyzing and optimizing deep networks by quantifying the flow and preservation of information across layers.

Mutual Information (MI) is a central concept in this framework, measuring the statistical dependence between input features and target outputs. A high MI value indicates that the extracted features retain critical information relevant to the task, which is particularly important in applications such as AD diagnosis, where subtle structural patterns in MRI data must be accurately captured. The Renyi Mutual Information between two random variables, X and Y, is defined as:

1 $$I_{\alpha } (X:Y) = \frac{1}{{\alpha - 1}}\log _{2} \left( {\sum\nolimits_{{x,y}} {P(X,Y} )\left( {\frac{{P(X,Y)}}{{P(X)P(Y)}}} \right)^{{\alpha - 1}} } \right)$$

Where P(X, Y) denotes the joint probability distribution, and P(X) and P(Y) represent the marginal distributions of X and Y, respectively. Analyzing MI across different layers of a neural network provides insights into how effectively the model transmits relevant information and highlights points where information loss or distortion may occur, guiding more effective architectural design.

In addition to MI, divergence measures such as Renyi Divergence play a crucial role in assessing the alignment between predicted and true data distributions. Renyi Divergence, defined for a parameter α ≥ 0, quantifies the discrepancy between two probability distributions and generalizes several classical divergence measures, offering flexibility in evaluating model robustness.

2 $$D_{\alpha } (P(X)||Q(X)) = \frac{1}{{\alpha - 1}}\log _{2} \left( {\sum\limits_{X} {P(X)} ^{\alpha } Q(X)^{{1 - \alpha }} } \right)$$

In this context, P(X) denotes the feature distribution derived from MRI data, while Q(X) represents the predicted probability distribution across the diagnostic categories—AD, MCI, and NC. Renyi Divergence offers a versatile approach to quantify the discrepancy between these distributions, with the parameter α adjusting the sensitivity to distributional differences.

Notably, when α = 1, Renyi Divergence reduces to the well-known Kullback–Leibler (KL) Divergence, a standard measure of information loss. Although divergence-based objectives can be incorporated into training or regularization in other deep-learning settings, Renyi divergence in the present study was used only as a post-hoc evaluation measure. It was computed after model training to quantify the discrepancy between learned output distributions and diagnostic class structure, thereby complementing conventional performance metrics by assessing output-distribution alignment. This rigorous alignment is critical in clinical contexts, where misclassification can lead to significant diagnostic and therapeutic consequences.

The Renyi order parameter α was set to 2, a commonly used value that offers a practical trade-off between estimation stability and sensitivity to higher-order statistical dependencies.

Previous studies, including our prior work on information-theoretic analysis of hierarchical temporal memory models^21^, have demonstrated that this choice provides reliable non-parametric estimation in high-dimensional feature spaces, particularly in medical imaging applications where sample size is limited and feature distributions are complex.

Another important divergence measure employed in this study is the Henze–Penrose Divergence (HP Divergence), a non-parametric method designed to quantify dissimilarities between two probability distributions, particularly in high-dimensional spaces. Unlike Renyi Divergence, which requires specifying a sensitivity parameter α, HP Divergence operates without any parametric assumptions, making it especially advantageous for comparing distributions of complex datasets.

The HP Divergence between two distributions, P and Q, is defined as:

3 $$D_{{HP}} (P(X)||Q(X)) = \frac{1}{{4ab}}\left( {\sum\limits_{x} {\frac{{\left( {aP(X) - bQ(X)} \right)^{2} }}{{aP(X) + bQ(X)}} - (a - b)^{2} } } \right)$$

In Eq. (3), P(X) and Q(X) denote the probability distributions over the feature space, while a and b represent the relative sample sizes drawn from distributions P and Q, respectively. This formulation captures the distributional differences between predicted and actual data, offering valuable insights into the alignment of model outputs with true underlying structures. Henze–Penrose Divergence (HP Divergence) is particularly well-suited for detecting subtle discrepancies in high-dimensional datasets, such as MRI images, where minor variations can significantly affect classification outcomes.

Integrating these information-theoretic concepts into the design and evaluation of hybrid architectures, such as the proposed CNN-LSTM framework, enables a more rigorous assessment of intra-slice spatial information retention and ordered inter-slice representation stability. By leveraging Mutual Information, Renyi Divergence, and HP Divergence, this study establishes a comprehensive and mathematically grounded framework for model evaluation. These measures provide complementary quantitative descriptions of representation behavior and output-distribution alignment, helping to bridge conventional predictive metrics with post-hoc interpretability analysis.

Despite the growing interest in deep learning architectures for Alzheimer’s disease classification, the majority of existing studies employ information-theoretic concepts primarily as optimization tools or theoretical analyses, rather than as systematic evaluation criteria. To the best of our knowledge, relatively few studies have investigated the use of information-theoretic measures as a unified post-hoc evaluation framework for assessing how effectively deep models preserve diagnostically relevant information in neuroimaging applications.

In the following sections, these metrics are systematically applied to quantify information preservation and divergence within the proposed CNN-LSTM architecture (see sections “Information-theoretic evaluation of the CNN-LSTM model” and “Primary reporting protocol”– “Evaluating model robustness through K-fold cross-validation”).

## Proposed method

Although CNN–LSTM architectures have been used to combine intra-slice spatial feature extraction with ordered inter-slice dependency modeling, their performance is commonly evaluated using classification accuracy alone. In this study, the emphasis is not placed on architectural novelty, but rather on investigating how effectively the hybrid model learns discriminative representations when assessed through information-theoretic criteria.

**Fig. 2:**
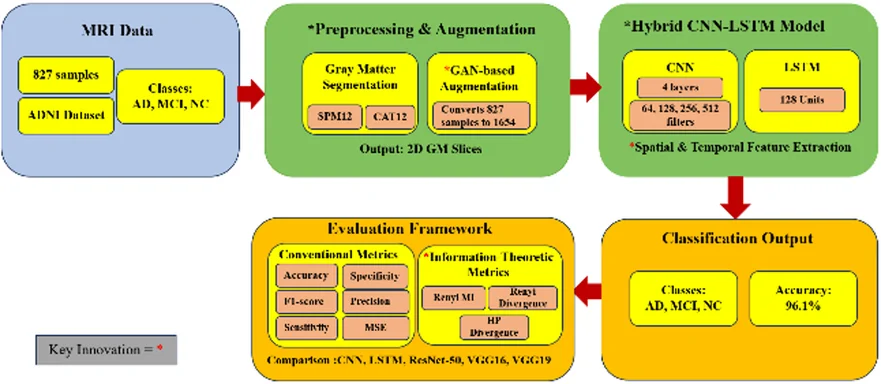
Overview of the proposed MRI classification and post-hoc evaluation workflow. Subject-level splitting was performed before preprocessing and augmentation to reduce data-leakage risk. GAN-generated samples were used only during training, and information-theoretic measures were applied after CNN–LSTM classification.

This study focuses on classifying Alzheimer’s disease (AD), mild cognitive impairment (MCI), and normal controls (NC) using brain MRI data. The analysis utilizes the Alzheimer’s Disease Neuroimaging Initiative (ADNI) dataset, comprising 827 subjects (NC: 340, MCI: 307, AD: 180) with 3D T1-weighted MRI scans. The dataset was randomly partitioned at the subject level into training (70%), validation (15%), and testing (15%) subsets to prevent data leakage across MRI slices. To strictly prevent any form of data leakage, all data partitioning was performed at the subject level prior to any preprocessing, slice extraction, or data augmentation steps. Specifically, MRI volumes from each subject were assigned exclusively to either the training, validation, or test set, ensuring that no slices from the same individual appeared across multiple subsets. An overview of the proposed algorithm is presented in Fig. 2.

### Ethics approval, informed consent, and regulatory compliance

This study used de-identified, previously collected structural MRI data obtained from the Alzheimer’s Disease Neuroimaging Initiative (ADNI) database under the applicable ADNI data-use policies. The ADNI study protocols were approved by the Institutional Review Board of the University of Southern California (USC IRB) and by the institutional review boards/ethics committees of the participating ADNI clinical sites. Written informed consent was obtained from all participants and/or their legally authorized representatives by the ADNI investigators at the participating sites prior to enrollment.

The present study was limited to secondary analysis of de-identified ADNI data. The authors did not recruit new participants, collect new human-subject data, contact participants, perform any intervention, access identifiable personal information, or redistribute participant-level data. No additional participant recruitment or informed-consent procedure was undertaken by the authors for the present secondary analysis. All methods used in the present study were performed in accordance with the relevant guidelines and regulations, including the Declaration of Helsinki, Good Clinical Practice guidelines, applicable institutional regulations, and ADNI data-use policies.

### Preprocessing and segmentation

Preprocessing was performed using the CAT12 and SPM12 toolboxes within the MATLAB R2019a environment. Following the default CAT12 pipeline, 3D T1-weighted MRI scans underwent affine and non-linear registration to align all images to a standardized template. Bias field correction was applied to mitigate intensity inhomogeneities commonly observed in MRI data.

Subsequently, images were segmented into three primary tissue classes: white matter (WM), gray matter (GM), and cerebrospinal fluid (CSF), with the analysis focused on GM. To ensure spatial consistency across subjects, segmented images were normalized to the Montreal Neurological Institute (MNI) space using the DARTEL (Diffeomorphic Anatomical Registration through Exponentiated Lie Algebra) algorithm. To minimize preprocessing-related information leakage, all subject-level splits were defined before preprocessing, slice extraction, model training, and GAN-based augmentation. CAT12/SPM12 preprocessing used fixed tissue-probability maps and standard MNI-space priors/templates; no study-specific DARTEL template, normalization template, intensity-normalization statistic, scaling parameter, or preprocessing model was estimated from the complete dataset or fitted using validation/test subjects. Validation and test images were processed with the same predefined settings and used only for model validation and final evaluation. Quality assurance was conducted by computing an automated quality control (QC) index via CAT12, supplemented by visual inspection to verify preprocessing integrity.

### Data augmentation using GAN

To improve the generalization capability of the proposed CNN-LSTM model, Generative Adversarial Networks (GANs) were employed for data augmentation. Given the inherent variability in brain structures and MRI acquisition conditions, training deep models on limited datasets can compromise performance.

Accordingly, a Deep Convolutional GAN (DCGAN) was utilized to generate synthetic MRI samples for each diagnostic category independently. As a result, the dataset was expanded from 827 subjects (NC: 340, MCI: 307, AD: 180) to 1654 subjects (NC: 680, MCI: 614, AD: 360), effectively doubling the number of training samples while preserving the original class distribution. Because GAN augmentation was applied proportionally across the three diagnostic classes, the original class distribution was preserved rather than fully equalized. Therefore, GAN augmentation was used primarily to mitigate data scarcity and increase training diversity, not as a complete class-balancing strategy. No class-weighted loss, balanced mini-batch sampling, or cost-sensitive learning strategy was used in the present implementation. To monitor potential class-related bias, we reported sensitivity, specificity, precision, and F1-score in addition to overall accuracy, and these metrics were averaged across subject-level cross-validation folds. The Generative Adversarial Network (GAN) was trained exclusively using MRI data from the training subset only. No images from the validation or test sets were used during GAN training under any circumstance. Synthetic MRI samples generated by the GAN were added solely to the training data to enhance model generalization and improve training diversity. Consequently, neither synthetic images nor any information derived from the validation or test subjects were involved in the training process, thereby eliminating potential information leakage and ensuring a fair and unbiased evaluation.

This augmentation strategy increased data diversity, mitigated overfitting, and improved the robustness of the classification model.

The GAN architecture comprised a generator network built with transposed convolutional layers, batch normalization, and ReLU activation, while the discriminator network utilized standard convolutional layers with Leaky ReLU activation to differentiate real from synthetic images. Adversarial training was conducted using the binary cross-entropy loss function, with optimization performed via the Adam algorithm (learning rate = 0.0002, batch size = 64). The model was trained for 100 epochs on an NVIDIA RTX 3090 GPU with CUDA acceleration to ensure computational efficiency.

To evaluate the quality and plausibility of the GAN-generated MRI samples, we used a combination of quantitative and qualitative assessments. Quantitatively, the generated images achieved a Structural Similarity Index (SSIM) of 0.92, indicating a high degree of structural similarity to real MRI scans. In addition, histogram-based comparison of pixel-intensity distributions showed close alignment between real and synthetic images, suggesting that the generated samples preserved the overall intensity characteristics of the training data.

Because conventional Fréchet Inception Distance (FID) is based on feature representations from natural-image classifiers and may not optimally reflect anatomical realism in structural MRI, FID was not used as the primary image-quality indicator in the revised analysis. Instead, SSIM and intensity-distribution analysis were used as the main quantitative checks. In addition to these quantitative assessments, generated samples were qualitatively reviewed by radiologists and researchers with neuroimaging experience to screen for obvious anatomical distortions, unrealistic intensity patterns, or class-inconsistent structures. This qualitative review did not reveal gross anatomically implausible features in the inspected samples. The review was used only as a supplementary plausibility check and was not used for model training, validation, test-set selection, or performance reporting. In the 10-fold comparative evaluation, incorporating GAN-generated samples into the training set improved the CNN–LSTM model’s accuracy from 94.9% to 96.7%, F1-score from 90.3% to 92.1%, sensitivity from 88.6% to 90.4%, and precision from 91.9% to 93.9%. The mean squared error also decreased from 0.020 to 0.014, indicating improved predictive stability under the present ADNI-based evaluation protocol. These results suggest that GAN-based augmentation contributed to improved generalization in the current experimental setting. However, because synthetic images may introduce distributional artifacts or fail to fully capture real-world pathological variability, these findings should be interpreted cautiously and require further validation on independent multi-center datasets. A detailed comparative analysis of the model’s performance with and without synthetic augmentation is presented in section “Comparative evaluation with state-of-the-art models”.

### Transformation of 3D MRI data into anatomically ordered 2D slice sequences

To reduce computational complexity while retaining anatomical continuity, each preprocessed 3D gray-matter MRI volume was transformed into a sequence of 2D sagittal slices arranged according to their anatomical order. This representation enables the CNN component to extract intra-slice spatial features while allowing the LSTM component to model dependencies across neighboring slices. Importantly, the resulting slice sequence should not be interpreted as a temporal sequence; rather, it represents ordered spatial positions within a registered brain volume.

The use of 2D slice sequences provides a computationally efficient alternative to full 3D processing, particularly in limited-sample medical imaging settings. Nevertheless, this representation may not capture all volumetric relationships available in the original 3D MRI scans. We therefore interpret the CNN–LSTM design as a pragmatic compromise between slice-independent 2D CNNs and more computationally demanding 3D CNN or transformer-based volumetric models. Future work should directly compare these alternatives under harmonized preprocessing and validation protocols.

In the present implementation, sagittal slices were ordered according to their anatomical position after spatial normalization.

Alternative slice-ordering strategies, such as interleaved or bidirectional slice sequences, and non-adjacent skip connections were not evaluated in the present study, as they were beyond its scope. Future work should investigate whether such mechanisms can improve long-range inter-slice dependency modeling and representation stability.

### Handcrafted features for statistical characterization and CNN–LSTM input preparation

To support clinical interpretability and statistical characterization of disease-related imaging patterns, a set of handcrafted structural and textural features was extracted from the preprocessed gray-matter MRI data. These features were not used as inputs to the CNN–LSTM classifier. Instead, they were analyzed separately to provide complementary information about structural and textural differences among AD, MCI, and NC groups.

Structural features included gray-matter volume in AD-relevant regions, including the hippocampus, entorhinal cortex, parahippocampal cortex, and medial temporal lobe, as well as cortical thickness in the posterior cingulate cortex, frontal lobe, and medial temporal lobe. Gray-matter volume was calculated from the SPM12 segmentation outputs, while cortical thickness was estimated using the CAT12 surface-based morphometry pipeline.

Textural features included contrast, homogeneity, entropy, and energy, computed using the gray-level co-occurrence matrix (GLCM). The GLCM was configured with a pixel distance of 1 and evaluated at angles of 0°, 45°, 90°, and 135° to characterize local gray-matter texture patterns.

A total of eleven handcrafted structural and textural features were selected based on their established relevance in prior Alzheimer’s disease research^12,35,36^. These handcrafted features were used only for statistical characterization and clinical interpretability analyses and were not provided to the CNN–LSTM model during training, validation, or testing. The CNN–LSTM model was trained end-to-end directly on preprocessed gray-matter image slices, enabling automatic learning of discriminative image-based representations. This separation was maintained to avoid introducing handcrafted-feature bias into the end-to-end classification pipeline.

### Proposed CNN-LSTM hybrid architecture for AD classification

The proposed classification framework uses a hybrid CNN–LSTM architecture that combines CNN-based intra-slice feature extraction with LSTM-based modeling of ordered inter-slice dependencies. In this design, CNN layers extract spatial features from individual preprocessed MRI slices, while LSTM layers process the resulting ordered slice-level representations to incorporate anatomical continuity across neighboring slices. The architecture should therefore be interpreted as a hybrid 2D slice-sequence model rather than a full volumetric model. Similarly, Huang et al.^37^ demonstrated the broader applicability of recurrent architectures for modeling structured dependencies in neuroimaging data. The convolutional component comprises four layers with 64, 128, 256, and 512 filters, respectively, each followed by ReLU activation to optimize feature extraction. Carefully selected kernel sizes ensure the convolutional layers effectively identify intricate spatial patterns. To enhance computational efficiency, two max-pooling layers are applied after every pair of convolutional layers, reducing the dimensionality of feature maps while preserving essential information.

Although MRI slices do not represent temporal evolution in the conventional sense, they exhibit an ordered spatial structure along anatomical axes. In this study, the LSTM component is employed not to model time-series dynamics, but to capture structured inter-slice dependencies and spatial continuity across consecutively ordered MRI slices. The LSTM component, equipped with 128 units, processes the extracted slice-level features to model ordered inter-slice dependencies. This integrated design enables robust feature extraction and reliable classification performance. The architecture culminates in a fully connected (FC) layer with three output neurons, corresponding to the diagnostic categories: AD, MCI, and NC.

**Fig. 3:**
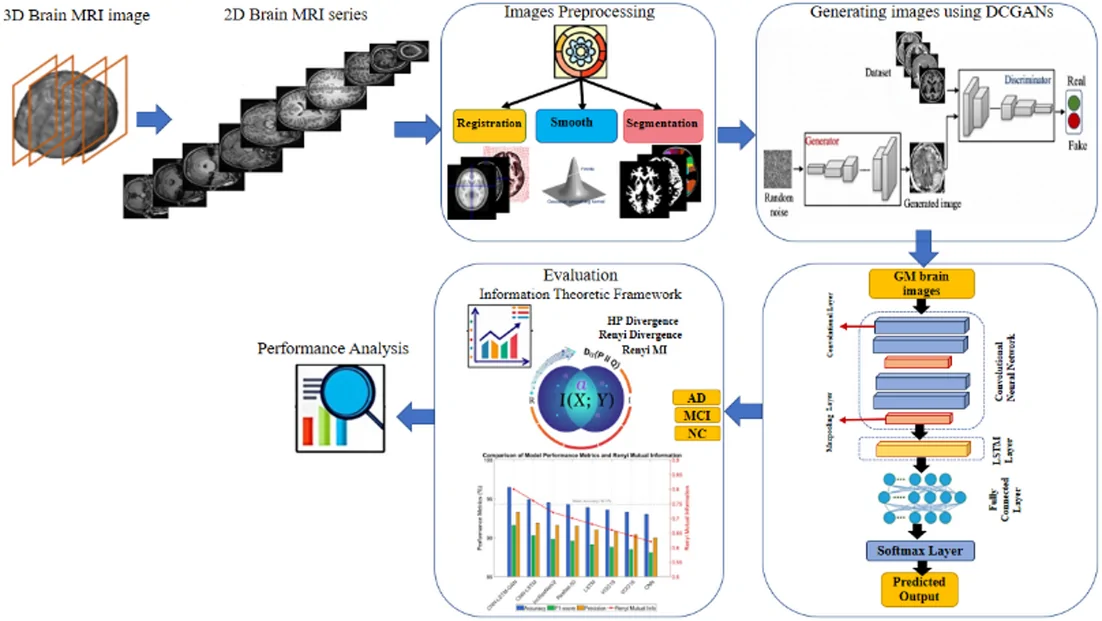
CNN–LSTM slice-sequence architecture for three-class AD/MCI/NC classification. CNN layers extract intra-slice spatial features from preprocessed gray-matter MRI slices, the LSTM component models anatomically ordered inter-slice dependencies rather than temporal progression, and fully connected layers perform final multi-class classification.

To promote generalization and prevent overfitting, dropout regularization with a rate of 0.1 is applied to the FC layers. The model is optimized using the Adam optimizer, configured with an initial learning rate of 0.0005 and adaptive learning rate decay to ensure stable and efficient convergence. This configuration was selected based on validation performance and was subsequently evaluated under the cross-validation protocol. Following the final convolutional layer, the LSTM layers enhance the classification process by modeling structured inter-slice dependencies among the extracted spatial features. The hybrid CNN-LSTM architecture is tailored to classify AD, MCI, and NC, leveraging all MRI slices concurrently. By effectively capturing both intra-slice spatial patterns and ordered inter-slice relationships, the model achieves a classification accuracy of 96.7%. The system was trained for 70 epochs using the Adam optimizer^38^ with an initial learning rate of 0.0005, ensuring robust and efficient convergence. The CNN–LSTM model was compared with benchmark architectures, including CNN, LSTM, ResNet-50, Inception-ResNet-V2, VGG16, and VGG19, under the same experimental protocol. These comparisons were used to assess whether combining intra-slice spatial feature extraction with ordered inter-slice dependency modeling provides measurable benefits over the evaluated baseline models. The overall structure of the proposed CNN–LSTM hybrid architecture is illustrated in Fig. 3.

### Information-theoretic evaluation of the CNN-LSTM model

To rigorously evaluate the performance of the proposed CNN-LSTM model beyond traditional accuracy-based metrics, three information-theoretic measures are employed: Renyi Mutual Information (MI), Renyi Divergence, and Henze-Penrose Divergence (HP Divergence). These metrics, detailed in Section II.C, provide complementary quantitative measures for assessing information preservation, representation stability, and output-distribution alignment in AD, MCI, and NC classification.

The input feature distribution, P(x), is estimated using Kernel Density Estimation (KDE) with a Gaussian kernel, ensuring robust representation of the data’s statistical properties. The output distribution, Q(x), is derived from the softmax layer of the CNN-LSTM model, computed as an average over 1000 Monte Carlo samples to enhance stability and reliability. These metrics quantify the model’s capacity to preserve critical information and distinguish between diagnostic categories, offering a nuanced perspective on its generalization and discriminative power.

- Renyi Mutual Information (MI) quantifies the statistical relationship between input features and target labels, providing insight into the relevance of extracted features. A higher MI score reflects the model’s ability to effectively capture diagnostic patterns critical for classification tasks.
- Renyi Divergence measures the discrepancy between input and output distributions, with lower values indicating closer alignment and minimal information loss, ensuring robust feature transformation.
- Henze-Penrose Divergence (HP Divergence) offers a non-parametric evaluation of distributional similarity, particularly effective in high-dimensional data settings where conventional parametric assumptions may be unreliable.

Experimental evaluation, performed with an optimized configuration (dropout rate of 0.1, learning rate of 0.0005), demonstrates that the CNN-LSTM model effectively captures task-relevant information. A Renyi Mutual Information (MI) score of 0.85 reflects a strong alignment between extracted features and true class labels, underscoring the model’s ability to identify diagnostically significant patterns. Furthermore, low Renyi Divergence (0.15) and Henze-Penrose Divergence (0.10) values indicate minimal information distortion, confirming that the model preserves structural fidelity and representational integrity during classification.

These findings suggest that the information-theoretic measures provide complementary evidence regarding representation stability and output-distribution alignment. However, these metrics should not be interpreted as standalone clinical indicators. Rather, they are used in this study as post-hoc analytical tools to support model comparison and interpretation alongside conventional classification metrics.

The proposed information-theoretic measures are not incorporated into the model’s loss function and do not influence parameter optimization during training. Instead, they are applied as post-hoc analytical tools to evaluate how effectively learned representations preserve diagnostically relevant information and maintain alignment between input and output distributions.

This analytical approach provides interpretable insights into model behavior, highlighting how learned representations align with critical diagnostic features in MRI data.

## Proposed method evaluation and results

### Primary reporting protocol

To avoid ambiguity in performance reporting, we define the subject-level 10-fold cross-validation results as the primary reporting protocol of this study. Therefore, the main performance of the proposed CNN–LSTM framework is reported as the mean ± standard deviation across cross-validation folds. Results obtained during hyperparameter tuning, including the 97.8% accuracy observed for the best dropout rate, learning rate, and convolutional-layer configuration, are treated as secondary configuration-level analyses rather than the primary estimate of model performance. Accordingly, the primary reported accuracy of the proposed model is 96.7%, as obtained from the cross-validation protocol.

### Evaluation metrics overview

To comprehensively assess the effectiveness of the proposed CNN-LSTM architecture, we adopted a dual-layer evaluation strategy that integrates both classical performance metrics and information-theoretic analysis. Standard indicators—such as accuracy, precision, sensitivity, specificity, F1-score, and mean squared error (MSE)—were utilized to quantify the model’s predictive accuracy. However, these conventional metrics may not fully reflect the model’s capacity to retain, transform, and transmit diagnostically relevant information.

To address this gap, we implemented an information-theoretic evaluation framework that offers a complementary lens for analyzing model behavior. Specifically, we applied Renyi Mutual Information (MI), Renyi Divergence, and Henze–Penrose Divergence (HPD) to investigate the internal information dynamics of the CNN-LSTM network. These measures enabled us to capture the depth and structure of the information flow beyond what standard metrics can reveal.

In addition, a rigorous evaluation protocol was employed, incorporating a confusion matrix to examine classification.

performance and k-fold cross-validation to ensure model stability and generalizability across data partitions. The following sections provide an in-depth discussion of these metrics and their implications for assessing the robustness and diagnostic precision of the proposed model.

### Confusion matrix and key performance metrics

The confusion matrix^39^ is a fundamental tool for assessing the performance of classification models. It organizes predictions into four categories: True Positives (TP), True Negatives (TN), False Positives (FP), and False Negatives (FN). These categories provide a clear framework for evaluating a model’s ability to correctly classify instances. By analyzing these values, critical performance metrics can be derived to quantify the model’s accuracy, precision, and overall reliability in distinguishing between classes.

To assess the performance of the proposed CNN-LSTM model, six key metrics were calculated, each providing unique insights into the model’s classification capabilities:

- Accuracy: Reflects the proportion of correctly classified instances across all cases, offering a broad measure of overall model performance.
- Sensitivity: Gauges the model’s ability to accurately detect positive cases (e.g., Alzheimer’s disease), with higher values indicating fewer missed diagnoses (false negatives).
- Specificity: Measures the model’s effectiveness in correctly identifying negative cases (e.g., normal controls), minimizing incorrect positive predictions (false positives).
- Precision: Quantifies the proportion of true positive predictions among all instances classified as positive, ensuring that positive classifications are reliable and reducing false positives.
- F1-score: Balances precision and sensitivity, providing a robust metric for evaluating performance on imbalanced datasets where one class may be underrepresented.
- Mean Squared Error (MSE): Captures the average squared difference between predicted and actual classifications, emphasizing prediction confidence. By penalizing larger deviations more heavily, MSE complements traditional metrics and highlights the model’s stability and reliability.

These metrics collectively offer a comprehensive evaluation of the model’s diagnostic accuracy and robustness, particularly for critical applications such as Alzheimer’s disease classification.

The mathematical formulations for the performance metrics are detailed in Eqs. (4) through (8).

As reported in the subsequent results tables, these metrics provide complementary views of model performance under the present ADNI-based evaluation protocol. The corresponding results support the stability of the proposed CNN–LSTM framework across the evaluated experimental settings, while clinical applicability requires further validation on independent and prospective datasets.

4 $$sensitivity = \frac{a}{{a + c}}$$

5 $$Accuracy = \frac{{a + d}}{{a + c + d + b}}$$

6 $$F_{1} - score = \frac{{2a}}{{2a + b + c}}$$

7 $$Specificity = \frac{d}{{b + d}}$$

8 $$Precision = \frac{a}{{a + b}}$$

### Impact of hyperparameters: dropout and learning rate

To optimize the performance and generalization of the proposed CNN-LSTM model, comprehensive hyperparameter tuning was performed, with a focus on the dropout rate and learning rate. These parameters are pivotal in regulating model complexity and preventing overfitting, thereby enhancing the model’s robustness and adaptability across diverse datasets.

1. *Dropout rate analysis*.

Dropout is a regularization method that randomly deactivates a subset of neurons during training, thereby reducing overfitting.

**Fig. 4:**
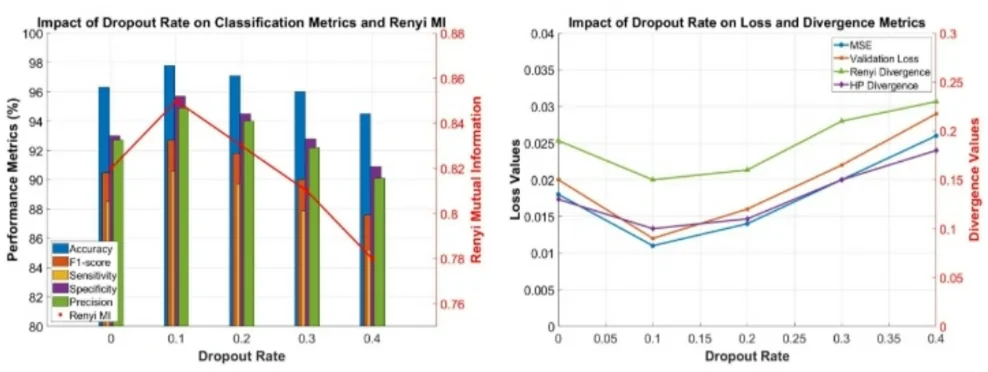
Effect of dropout rate on CNN–LSTM classification performance and post-hoc information-theoretic metrics. A dropout rate of 0.1 provided the most favorable balance between predictive performance, validation loss, mutual information, and divergence measures, whereas higher dropout rates were associated with reduced performance and increased divergence.

To identify the optimal dropout rate, an ablation study was conducted using a predefined uniform search grid, where the dropout rate was varied within the set $\left\{ {0.0,0.1,0.2,0.3,0.4} \right\}$. This grid was selected to provide systematic coverage of the regularization spectrum while maintaining computational efficiency. As summarized in Table 1; Fig. 4. a dropout rate of 0.1 achieved the highest accuracy (97.8%) and F1-score (92.7%), indicating a well-generalized model.

**Table 1:** Performance of the CNN-LSTM method at various dropout rates.

Dropout rate	Accuracy (%)	F1-Score (%)	Sensitivity (%)	Specificity (%)	Precision	MSE	Validation loss	Renyi MI	Renyi Div	HP Div
0	96.3	90.5	88.5	93.0	92.7	0.018	0.020	0.82	0.19	0.13
**0.1**	**97.8**	**92.7**	**90.6**	**95.7**	**94.9**	**0.011**	**0.012**	**0.85**	**0.15**	**0.10**
0.2	97.1	91.8	89.7	94.5	94.0	0.014	0.016	0.83	0.16	0.11
0.3	96.0	90.0	87.9	92.8	92.2	0.020	0.022	0.81	0.21	0.15
0.4	94.5	87.6	85.2	90.9	90.1	0.026	0.029	0.78	0.23	0.18

Significant values are in [bold].

Lower dropout rates (e.g., 0) resulted in noticeable overfitting, evidenced by a substantial gap between training and validation performance.

All dropout configurations were trained under identical conditions using an early stopping strategy based on validation loss, where training was terminated if no improvement was observed for 10 consecutive epochs. The model checkpoint corresponding to the minimum validation loss was retained to ensure fair and well-converged comparisons across configurations. In contrast, higher dropout rates (≥ 0.3) resulted in excessive information loss, reducing both sensitivity and specificity.

The role of information-theoretic measures provided additional clarity regarding the model’s behavior under varying regularization settings. When the dropout rate was set to 0.1, the model achieved its highest Renyi Mutual Information (MI) value of 0.85, indicating that this configuration enabled the extraction of features most relevant to the classification task. This finding aligns with the concurrent improvements observed in both accuracy and F1-score, underscoring the value of moderate dropout in maintaining informative representations.

Conversely, as the dropout rate increased beyond this point, a gradual rise in Renyi Divergence and Henze–Penrose Divergence (HPD) was observed. These trends suggest growing discrepancies between the predicted outputs and true data distributions—signs of deteriorating alignment and information loss due to overly aggressive regularization.

Among the tested configurations, the 0.1 dropout rate consistently delivered the most favorable outcomes, as evidenced in Table 1. It achieved the lowest mean squared error (MSE) at 0.011 and preserved the highest mutual information. Lower dropout rates, such as 0, led to overfitting, reflected by an increased validation loss (0.020), while higher rates (e.g., ≥ 0.3) caused underfitting, likely due to excessive suppression of useful features. These results collectively affirm that a dropout rate of 0.1 strikes an optimal balance—enhancing generalization without compromising the model’s capacity to retain discriminative information.

2) *Learning rate analysis*.

To determine the optimal trade-off between convergence speed and training stability, three learning rates—0.005, 0.0005, and 0.00005—were evaluated. To analyze the effect of the learning rate on model performance, a controlled ablation study was conducted under identical training conditions. For all configurations, the model was trained using the same architecture, batch size, and optimization settings, with early stopping applied based on validation loss. The optimal learning rate was selected according to the configuration that achieved the lowest validation loss while maintaining stable validation accuracy, ensuring a fair and consistent comparison across tested values.

All other hyperparameters were kept fixed, allowing the isolated assessment of the learning rate’s impact on convergence behavior and generalization.

As presented in Table 2. a learning rate of 0.0005 yielded the best overall performance, achieving an accuracy of 97.8% and an F1-score of 92.7%. A higher learning rate (0.005) led to instability in the training process, manifested as oscillations in the loss function and reduced generalization capability. Conversely, a lower learning rate (0.00005) resulted in slower convergence and only marginal improvements in accuracy. In contrast, the intermediate learning rate of 0.0005 offered an ideal trade-off, promoting both reliable convergence and reduced overfitting.

**Table 2:** Performance of the CNN-LSTM method with different learning rates.

Learning rate	Accuracy (%)	F1-Score (%)	Sensitivity (%)	Specificity (%)	Precision (%)	MSE	Validation Loss	Renyi MI	Renyi Div	HP Div
0.005	95.0	88.3	86.4	91.2	90.2	0.023	0.025	0.80	0.21	0.15
**0.0005**	**97.8**	**92.7**	**90.6**	**95.7**	**94.9**	**0.011**	**0.012**	**0.85**	**0.15**	**0.10**
0.00005	96.1	90.4	88.3	93.0	92.6	0.016	0.018	0.82	0.18	0.14

Significant values are in [bold].

The learning rate of 0.0005 yielded the most favorable outcomes, with the model achieving a peak Renyi Mutual Information (MI) score of 0.85. This high MI value indicates that the model effectively preserved features most relevant to the classification task, contributing directly to its enhanced predictive performance.

In contrast, a higher learning rate (0.005) led to greater misalignment between predicted and true distributions, as reflected by elevated Renyi and Henze–Penrose divergence values. While the lowest tested rate (0.00005) produced marginally lower divergence, it also resulted in a reduced MI, suggesting a decline in the informativeness of extracted features and diminished discriminative capacity.

These observations highlight the pivotal influence of learning rate tuning in balancing information retention with model convergence and generalization. The consistency between conventional evaluation metrics—such as accuracy, F1-score, and mean squared error—and information-theoretic indicators further supports the robustness of the CNN-LSTM model under the 0.0005 learning rate configuration.

As detailed in Table 2, this setting yielded the highest classification scores and the lowest MSE (0.011), confirming its effectiveness. It enabled stable convergence while avoiding the volatility associated with higher rates and the learning inefficiencies of lower ones. The convergence of peak MI values with minimized divergence underscores the suitability of this rate for reliable and interpretable Alzheimer’s disease classification.

3) *Effect of the number of convolutional layers*.

To evaluate the effect of convolutional layer count on the performance of the proposed CNN-LSTM model, we tested configurations ranging from one to seven layers. The layer-depth search was conducted using a predefined and exhaustive grid of $\left\{ {1,2,3,4,5,6,7} \right\}$ convolutional layers. This range was selected to systematically explore the trade-off between representational capacity and model generalization, while avoiding excessive architectural complexity. Shallow configurations may be insufficient to capture hierarchical spatial patterns in brain MRI data, whereas overly deep architectures can introduce redundant feature representations and increase the risk of overfitting, particularly in limited-sample medical imaging scenarios. Therefore, the selected search space was designed to identify an optimal balance between expressive feature extraction and stable learning behavior.

As presented in Table 3, the architecture with four convolutional layers yielded optimal performance, achieving an accuracy of 97.8%, an F1-score of 92.7%, and the lowest MSE of 0.011, demonstrating superior classification accuracy and stability.

Among the tested architectural configurations, the model with four convolutional layers proved most effective in capturing complex spatial patterns within MRI data. This configuration attained the highest Renyi Mutual Information (MI) score of 0.85, indicating a robust and informative feature representation that significantly enhanced the classification performance.

Shallower networks, utilizing only one or two convolutional layers, exhibited a marked reduction in sensitivity, likely due to their limited ability to capture subtle neuroanatomical variations associated with AD. Conversely, architectures with more than four layers showed diminishing returns, with evidence of feature redundancy and slight overfitting, indicating an optimal balance at four layers.

Information-theoretic metrics further corroborated these findings. The four-layer configuration consistently produced the lowest Renyi and Henze–Penrose divergence values, reflecting strong alignment between the model’s internal feature representations and the underlying data distribution. These results highlight the four-layer architecture’s ability to strike an optimal balance between expressive feature extraction and robust generalization, rendering it particularly well-suited for capturing the intricate spatial complexity of brain MRI scans.

**Table 3:** Performance of the CNN-LSTM Model with Varying Numbers of Convolutional Layers.

Number of convolution layers	Accuracy (%)	F1-Score (%)	Sensitivity (%)	Specificity (%)	Precision (%)	MSE	Validation loss	Renyi MI	Renyi Div	HP Div
1	92.0	85.5	83.5	89.0	87.6	0.040	0.044	0.74	0.30	0.22
2	94.5	88.8	86.8	91.5	90.9	0.028	0.031	0.78	0.25	0.18
3	96.0	90.5	88.5	93.2	92.6	0.018	0.020	0.81	0.20	0.14
**4**	**97.8**	**92.7**	**90.6**	**95.7**	**94.9**	**0.011**	**0.012**	**0.85**	**0.15**	**0.10**
5	97.3	91.8	89.7	94.8	94.0	0.013	0.015	0.83	0.17	0.12
6	96.7	90.9	88.8	93.9	93.1	0.016	0.018	0.80	0.19	0.14
7	95.9	89.7	87.5	92.6	92.0	0.021	0.024	0.77	0.22	0.17

Significant values are in [bold].

### Evaluating model robustness through K-fold cross-validation

To ensure the generalizability of the proposed CNN-LSTM model and mitigate the risk of overfitting, a 10-fold cross-validation strategy^40^ was implemented, a widely recognized method in machine learning. The dataset was evenly divided into ten subsets, with each subset used once as the test set while the remaining nine served as the training set. This iterative approach guaranteed that every sample contributed to both model training and evaluation, thereby bolstering the robustness and reliability of the performance results.

It should be emphasized that all cross-validation experiments were conducted using the optimal hyperparameter configuration identified in Tables 1, 2 and 3. Therefore, the results reported in Table 4 reflect averaged performance across different data partitions rather than the best-case performance obtained under a single evaluation setting. The cross-validation results, presented in Table 4, confirm the stability and robustness of the proposed CNN-LSTM model across different data partitions. In addition to reporting the mean performance, the standard deviation is provided for all evaluation metrics to explicitly quantify inter-fold variability. The consistently low standard deviation observed in accuracy, F1-score, specificity, and mean squared error demonstrates that the proposed model delivers stable and reliable performance under varying training–testing splits, highlighting its strong generalization capability.

Furthermore, the low variance in Renyi Mutual Information, Renyi Divergence, and Henze–Penrose Divergence across folds indicates consistent information preservation and stable alignment between input and output distributions, reinforcing the robustness of the learned representations. Although sensitivity showed moderate fold-wise variability, ranging from 88.00% to 92.39%, the model maintained stable overall accuracy, F1-score, specificity, MSE, and information-theoretic behavior across folds. This supports the robustness of the proposed CNN–LSTM framework under the subject-level cross-validation protocol.

**Table 4:** Results of the CNN-LSTM method using K-fold cross-validation.

Fold	Accuracy (%)	F1-score (%)	Sensitivity (%)	Specificity (%)	Precision (%)	MSE	Validation loss	Renyi MI	Renyi Div	HP Div
1	96.9	92.4	92.12	95.5	92.68	0.012	0.014	0.85	0.15	0.13
2	96.5	91.9	88.99	95.0	94.97	0.015	0.017	0.81	0.18	0.14
3	96.8	92.3	91.40	95.4	93.23	0.013	0.015	0.84	0.16	0.12
4	96.6	91.8	89.16	95.1	94.66	0.014	0.016	0.82	0.17	0.13
5	96.7	92.1	90.42	95.2	93.86	0.014	0.016	0.83	0.16	0.11
6	96.9	92.5	92.39	95.6	92.61	0.012	0.014	0.86	0.15	0.12
7	96.4	91.7	88.00	94.9	95.74	0.016	0.018	0.78	0.20	0.14
8	96.6	91.9	89.43	95.1	94.56	0.015	0.017	0.80	0.18	0.11
9	96.8	92.3	91.40	95.4	93.23	0.013	0.015	0.84	0.16	0.12
10	96.6	92.2	90.69	95.3	93.76	0.015	0.017	0.81	0.19	0.10
**Mean ± Std**	**96.68 ± 0.17**	**92.11 ± 0.27**	**90.40 ± 1.46**	**95.25 ± 0.23**	**93.93 ± 1.03**	**0.014 ± 0.001**	**0.016 ± 0.001**	**0.82 ± 0.02**	**0.17 ± 0.02**	**0.12 ± 0.01**

Significant values are in [bold].

Note: Values are reported as mean ± sample standard deviation across the 10 subject-level cross-validation folds. Percentage-based metrics in this table are reported with higher precision to avoid rounding-related ambiguity, whereas summary comparison tables report rounded values.

These results collectively affirm the robustness, generalization, and stability of the CNN-LSTM model across diverse data conditions. The incorporation of information-theoretic analysis provides a complementary perspective that enhances the interpretability and reliability of conventional performance metrics, supporting the stability of the model under the present ADNI-based evaluation protocol, while further external validation is required before any clinical interpretation.

### Comparison with recent Alzheimer’s studies

To contextualize the proposed framework within recent MRI-based Alzheimer’s disease classification studies, Table 5 summarizes representative directly related works, including recently suggested state-of-the-art methods^17–20,41,42^. This comparison is intended to provide literature context rather than to claim direct numerical superiority, because the included studies differ in dataset composition, class definitions, preprocessing pipelines, augmentation strategies, and validation protocols. Recent XRDNet and NeuroMixFormer studies further illustrate architecture-centered advances, including residual dense fusion, spatial context fusion, attention mechanisms, mixture-of-experts routing, and explainability^41,42^. In contrast, the present study focuses on post-hoc information-theoretic evaluation of learned CNN–LSTM representations. Therefore, the most reliable performance evidence for the present study is provided by the within-study benchmark comparisons reported in section “Comparative evaluation with state-of-the-art models”.

**Table 5:** Contextual comparison with directly related recent MRI-based Alzheimer’s disease classification studies.

Feature	Proposed method	Amin-Naji et al. (2019)^17^	Abbas et al. (2023)^18^	Folego et al. (2020)^19^	Ayus & Gupta (2024)^20^	Abbas et al. (2025) / XRDNet^41^	Abbas et al. (2026) / NeuroMixFormer^42^
Model architecture	CNN-LSTM (4 conv, 64–512, 128 LSTM, dropout 0.1)	Siamese CNN	JD-CNN (Jacobian- based)	3D-CNN (4 conv, 64–512)	Hybrid CNN-Conv1D- LSTM with hierarchical residual ensemble (HReENet)	Residual dense fusion network with SCF blocks	Multistage attention- enhanced MoE with dynamic routing
Dataset	ADNI; 827 subjects; training-only GAN augmentation	ADNI (~ 500, sMRI)	ADNI (~ 600, sMRI 3D)	ADNI (900, sMRI 3D)	Kaggle (~ 6400, sMRI 2D)	Falah/Alzheimer_MRI; 6,400 2D MRI images	ADNI, Kaggle Augmented Alzheimer’s MRI, Mendeley
Data augmentation	Yes (DCGAN, SSIM: 0.92)	No	No	No	Yes (geom, color)	Not specified	Kaggle augmented dataset used; additional aug. NR
Classes	AD, MCI, NC	AD, NC	AD, NC	AD, MCI, NC	ND, VMD, MID, MOD	ND, VMD, MID, MOD	Dataset-dependent AD classification/staging
Eval metrics	Acc: 96.7%, F1: 92.1%, Renyi MI: 0.82, HP Div: 0.12	Acc: 91.83%	Acc: ~94%, AUC: ~0.92	Acc: 91.5%, AUC: 0.90	Acc: 99.97%, F1: 99.97%	Acc: 96.5%	Acc: 99.86% ADNI, 99.48% Kaggle, 90.28% Mendeley
Info-theoretic evaluation	Yes (Renyi MI, Renyi Div, HP Div)	No	No	No	No	No	No
Explainability	High (MI, Div analysis)	Low	Moderate (Jacobian maps)	Low	Low	Yes; conformal prediction	Yes; routing/attention analysis
Limitations	ADNI-specific; single- modality; no external cohort validation	No explicit inter-slice dependency modeling, no aug	No explicit inter-slice dependency modeling, no aug	High comp cost, no aug	Different dataset and class definitions; limited interpretability	Different dataset/ classes; no post-hoc IT evaluation	High cost/time; different protocols; no post-hoc IT evaluation

Note: Values are not head-to-head comparisons because datasets, class definitions, preprocessing pipelines, augmentation strategies, and validation protocols differ. IT: information-theoretic; NR: not reported; MoE: mixture of experts; SCF: spatial context fusion.

### Comparative evaluation with state-of-the-art models

The results reported in Table 6 were obtained using the same optimal hyperparameter configuration identified in Tables 1, 2 and 3, with performance metrics averaged across 10-fold cross-validation. The slight variations observed compared with the best-case results reflect the averaging process across folds. Table 6 presents a detailed comparison of the proposed CNN–LSTM framework against the evaluated benchmark architectures, including standard CNN, LSTM, ResNet-50, Inception-ResNet-V2, VGG16, and VGG19 models. As shown in Table 6, the proposed method achieved higher performance than the evaluated benchmark architectures in terms of accuracy, F1-score, and information-theoretic measures under the same 10-fold cross-validation protocol.

The CNN–LSTM model achieved a mean classification accuracy of 96.7%, an F1-score of 92.1%, and the lowest mean squared error (MSE = 0.014) across the 10-fold cross-validation protocol. These outcomes suggest that the model benefits from combining intra-slice spatial feature extraction with inter-slice dependency modeling for MRI-based Alzheimer’s disease classification. In addition to conventional evaluation metrics, the CNN–LSTM model showed favorable information-theoretic behavior, with Renyi mutual information of 0.82 and lower divergence values than the evaluated benchmark models (Renyi divergence = 0.17; Henze–Penrose divergence = 0.12). Minor numerical differences between these values and those reported in Tables 1, 2 and 3 arise from cross-validation averaging and Monte Carlo estimation. Specifically, Tables 1, 2 and 3 report representative optimized runs, whereas Tables 4, 5 and 6 present mean performance across 10-fold cross-validation for comparative evaluation.

Compared with alternative architectures such as ResNet-50 and Inception-ResNet-V2, the proposed model showed higher mutual information and lower divergence values under the same experimental protocol, suggesting stronger information retention and closer output-distribution alignment. These findings suggest that combining convolutional spatial encoding with LSTM-based ordered inter-slice spatial dependency modeling can improve the representation of anatomically structured MRI slice sequences. Among the evaluated architectures, the CNN–LSTM model showed the most favorable combination of conventional performance metrics and information-theoretic indicators, suggesting improved alignment between learned representations and the diagnostic structure of the dataset.

The contribution of GAN-based augmentation was also assessed by comparing CNN–LSTM performance with and without synthetic training samples. As reported in Table 6, incorporating GAN-generated samples into the training set improved accuracy from 94.9% to 96.7%, F1-score from 90.3% to 92.1%, sensitivity from 88.6% to 90.4%, and precision from 91.9% to 93.9%, while reducing MSE from 0.020 to 0.014. These results suggest that GAN-based augmentation contributed to improved generalization under the present ADNI-based evaluation protocol. However, because synthetic images may introduce distributional artifacts, these findings should be interpreted with caution and require further validation using independent datasets. The statistical robustness of the primary CNN–LSTM results and the controlled comparative margins relative to benchmark architectures are further summarized in section “Statistical robustness and cComparative margin analysis”.

**Table 6:** Comparison of the CNN-LSTM with other cutting-edge models.

Method	Accuracy (%)	F1-score (%)	Sensitivity (%)	Specificity (%)	Precision (%)	MSE	Renyi MI	Renyi Div	HP Div
**Proposed** CNN- LSTM with GAN	**96.7**	**92.1**	**90.4**	**95.3**	**93.9**	**0.014**	**0.82**	**0.17**	**0.12**
Proposed CNN- LSTM without GAN	94.9	90.3	88.6	93.6	91.9	0.020	0.76	0.22	0.16
Inception-ResNet- V2	94.5	89.8	88.0	93.3	91.6	0.021	0.72	0.25	0.17
ResNet-50	94.3	89.6	87.8	93.1	91.5	0.022	0.70	0.27	0.18
LSTM	93.9	89.1	87.3	92.7	91.0	0.023	0.68	0.29	0.19
VGG19	93.6	88.8	86.9	92.4	90.8	0.024	0.66	0.30	0.20
VGG16	93.3	88.5	86.7	92.1	90.4	0.025	0.64	0.31	0.21
CNN	93.0	88.1	86.3	91.8	90.0	0.026	0.62	0.33	0.22

Significant values are in [bold].

Note: GAN-generated samples were added only to the training folds; validation and test folds contained only real MRI data.

Collectively, the results presented in Tables 1, 2, 3, 4, 5 and 6 suggest that the proposed CNN–LSTM framework achieves strong predictive performance while maintaining favorable information-theoretic behavior. The consistency observed across conventional performance metrics and information-theoretic evaluations supports the potential value of the framework for interpretable neuroimaging-based decision support, although prospective external validation is required before clinical deployment.

### Statistical robustness and comparative margin analysis

To improve the transparency of performance reporting, the primary CNN–LSTM results were analyzed using fold-wise statistics from the 10 subject-level cross-validation folds. For each metric, the mean, sample standard deviation (SD), standard error (SE), and 95% confidence interval (CI) were computed. Because the number of folds was limited, 95% CIs were estimated using the t-distribution with nine degrees of freedom. This analysis provides an estimate of inter-fold variability and avoids relying on a single point estimate of model performance.

The cross-validation robustness results are summarized in Table 7. The narrow confidence interval for accuracy indicates stable overall performance across folds, while the wider interval observed for sensitivity suggests that class-level detection performance remains more variable and should be further assessed in external validation studies. The information-theoretic measures also showed limited fold-wise variability, supporting the consistency of the representation-level behavior observed in the proposed framework.

For comparisons with previously published studies in Table 5, formal statistical significance testing was not performed because the studies differ in dataset composition, class definitions, preprocessing pipelines, augmentation strategies, and validation protocols. Therefore, these comparisons are interpreted only as literature context. In contrast, the benchmark architectures in Table 6 were evaluated within the present experimental protocol; therefore, their differences from the proposed CNN–LSTM model are reported as controlled comparative margins in Table 8. These margins provide practical effect estimates under the same evaluation setting without making unsupported cross-study statistical superiority claims.

**Table 7:** Fold-wise statistical robustness of the proposed CNN–LSTM model across 10 subject-level cross-validation folds.

Metric	Mean	SD	SE	CI 95%
Accuracy (%)	96.68	0.17	0.05	96.56–96.80
F1-score (%)	92.11	0.27	0.09	91.92–92.31
Sensitivity (%)	90.40	1.46	0.46	89.35–91.45
Specificity (%)	95.25	0.23	0.07	95.09–95.41
Precision (%)	93.93	1.03	0.33	93.19–94.67
MSE	0.0139	0.0014	0.0004	0.0129–0.0149
Validation loss	0.0159	0.0014	0.0004	0.0149–0.0169
Renyi MI	0.824	0.025	0.008	0.806–0.842
Renyi divergence	0.170	0.017	0.005	0.158–0.182
HP divergence	0.122	0.013	0.004	0.113–0.131

**Table 8:** Controlled comparative margins between the proposed CNN–LSTM model and benchmark architectures evaluated under the same experimental protocol.

Comparator	Δ Accuracy (%)	Δ F1-score (%)	Δ Sensitivity (%)	Δ Specificity (%)	Δ Precision (%)	MSE reduction	Renyi MI gain	Renyi Div reduction	HP Div reduction
CNN-LSTM without GAN	+ 1.8	+ 1.8	+ 1.8	+ 1.7	+ 2.0	0.006	+ 0.06	0.05	0.04
Inception-ResNet-V2	+ 2.2	+ 2.3	+ 2.4	+ 2.0	+ 2.3	0.007	+ 0.10	0.08	0.05
ResNet-50	+ 2.4	+ 2.5	+ 2.6	+ 2.2	+ 2.4	0.008	+ 0.12	0.10	0.06
LSTM	+ 2.8	+ 3.0	+ 3.1	+ 2.6	+ 2.9	0.009	+ 0.14	0.12	0.07
VGG19	+ 3.1	+ 3.3	+ 3.5	+ 2.9	+ 3.1	0.010	+ 0.16	0.13	0.08
VGG16	+ 3.4	+ 3.6	+ 3.7	+ 3.2	+ 3.5	0.011	+ 0.18	0.14	0.09
CNN	+ 3.7	+ 4.0	+ 4.1	+ 3.5	+ 3.9	0.012	+ 0.20	0.16	0.10

Positive values indicate improvement of the proposed model over the comparator; MSE and divergence values are reported as reductions.

Note: Comparative margins were calculated from the within-study benchmark results reported in Table 6. These values are descriptive effect margins under a shared experimental protocol and are not interpreted as formal inferential significance tests.

### Ablation study

An ablation-style comparison was conducted to examine the relative contributions of the CNN component, the LSTM component, and GAN-based augmentation. As shown in Table 6, the CNN-only model achieved 93.0% accuracy, while the LSTM-only model achieved 93.9%, indicating that each individual component captured only part of the relevant information. The CNN–LSTM model without GAN augmentation improved accuracy to 94.9%, suggesting that combining intra-slice spatial feature extraction with ordered inter-slice dependency modeling provided measurable benefit over either component alone. When GAN-generated samples were added only to the training folds, accuracy increased further to 96.7%, with corresponding improvements in F1-score, sensitivity, precision, MSE, and information-theoretic measures. These results suggest that both the hybrid CNN–LSTM design and training-only GAN augmentation contributed to the final performance. However, because the information-theoretic measures were used only as post-hoc evaluation tools and not as training components, their contribution should be interpreted as analytical rather than performance-generating.

### Computational considerations

Computational profiling was performed on a workstation equipped with an Intel Core i9-class CPU, 64 GB RAM, and a single NVIDIA RTX 3090 GPU with 24 GB VRAM. In a representative implementation run, offline DCGAN augmentation required approximately 2.1 h for 100 epochs, with peak GPU memory usage of approximately 9.2 GB. CNN–LSTM training required approximately 3.5 h for 70 epochs in a representative training fold, with peak GPU memory usage of approximately 8.4 GB. After preprocessing and excluding disk I/O, CNN–LSTM inference required approximately 14.2 ms per subject. The post-hoc information-theoretic analysis was computed only after inference on frozen representations and required approximately 41.5 s per evaluation run, with memory usage below 0.5 GB; therefore, it did not affect model optimization or backpropagation time. These results suggest that the proposed framework is computationally feasible on a single high-end GPU for offline decision-support research, although larger multi-center validation is required before prospective clinical deployment.

## Discussion

This study presents a CNN–LSTM-based framework that combines intra-slice spatial feature extraction with ordered inter-slice dependency modeling for classifying Alzheimer’s disease (AD), mild cognitive impairment (MCI), and normal controls (NC) using structural MRI data. With a mean subject-level cross-validation accuracy of 96.7%, the proposed model showed higher performance than the evaluated benchmark architectures, including ResNet-50, VGG16, standalone CNN, and LSTM models, under the same experimental protocol.

Recent medical imaging studies have explored attention mechanisms, feature fusion, transformer-based modeling, and mixture-of-experts routing to improve representation learning and predictive performance^28–31,41,42^. The present framework is complementary to these architecture-centered advances, because it focuses on evaluating information preservation, representation stability, and distributional alignment using post-hoc information-theoretic criteria beyond conventional accuracy-based assessment.

Unlike prior approaches, such as Payan and Montana^4^, which rely on 3D CNNs to capture volumetric patterns, or Sarraf and Tofighi^27^, which utilize basic CNNs for AD classification, our hybrid framework leverages LSTM layers to model structured inter-slice dependencies across ordered MRI slices. These results suggest that modeling ordered inter-slice dependencies may provide additional anatomical context beyond slice-independent representations under the present ADNI-based protocol. A key strength of this work lies in its use of information-theoretic measures—Renyi Mutual Information, Renyi Divergence, and Henze-Penrose Divergence—to evaluate model performance beyond traditional metrics. The model achieved a high Mutual Information score (0.82) and low divergence values (Renyi: 0.17, HPD: 0.12), indicating strong preservation of diagnostically relevant information. These metrics provide a transparent view of the model’s internal representations, addressing the interpretability demands of medical AI systems where trust and reliability are paramount. A key contribution of this study lies not in proposing a novel network architecture, but in shifting the focus of model assessment from purely outcome-driven metrics to information-centric evaluation. By explicitly quantifying how diagnostic information is retained and transformed across the learning process, the proposed framework provides a principled and interpretable basis for comparing deep learning models in clinical neuroimaging, where reliability and transparency are as critical as predictive accuracy. From a methodological perspective, information-theoretic measures play a critical role in validating both model design and parameter selection. While conventional metrics evaluate classification outcomes, mutual information and divergence-based measures quantify how effectively discriminative information is preserved and aligned throughout the learning process. The observed consistency between optimal hyperparameter configurations—such as moderate network depth and dropout rate—and favorable information-theoretic trends indicates that these design choices are not merely performance-driven, but also support stable and meaningful feature representations. This alignment reinforces the robustness and generalization capability of the proposed architecture, particularly in data-constrained medical imaging settings. Such methodological insights may be useful for research-oriented model auditing and reliability assessment, although they should not be interpreted as evidence of clinical readiness. It should be emphasized that the LSTM component captures inter-slice dependencies in ordered MRI slices, rather than modeling true temporal evolution. Despite its promising results, the study has notable limitations. First, the reliance on the ADNI dataset, while high-quality, limits generalizability due to its controlled acquisition settings and predominantly Western demographic. This may introduce biases, potentially reducing performance in diverse clinical populations. Second, although GAN-based data augmentation bolstered model robustness, synthetic images risk introducing distributional artifacts that may not fully align with real-world MRI variability. Although GAN-generated samples were qualitatively reviewed by radiologists and neuroimaging-experienced researchers, this review was not conducted as a formal blinded radiologist-reader study; future work should include structured radiologist scoring and independent validation of synthetic-image realism. Because GAN augmentation was applied proportionally across diagnostic groups, it improved training diversity but preserved the original class proportions rather than fully equalizing AD, MCI, and NC samples. Future work should examine class-weighted loss functions, balanced mini-batch sampling, and cost-sensitive learning strategies to further reduce potential class-related bias, particularly for the smaller AD class. Third, the exclusive use of structural MRI overlooks complementary modalities, such as PET or cognitive assessments, which could enrich diagnostic accuracy. To mitigate these, future efforts could validate the model on multi-center datasets, refine GAN-generated image fidelity, and integrate multimodal data to capture a broader spectrum of AD biomarkers. The present findings should therefore be interpreted as evidence of promising benchmark performance on ADNI structural MRI data rather than evidence of immediate clinical deployability. Before clinical translation, the framework requires validation on independent multi-center cohorts, assessment across heterogeneous scanner protocols, prospective evaluation, and comparison with radiologist- or neurologist-assisted diagnostic workflows. In this context, the proposed information-theoretic measures may be useful as supplementary tools for model auditing and reliability assessment, but they should not be treated as standalone clinical decision indicators. Future work should also investigate multimodal integration, including PET, cognitive scores, genetic markers, and longitudinal imaging, to better capture the heterogeneous nature of Alzheimer’s disease progression. The with-versus-without GAN analysis suggests that the hybrid CNN–LSTM design contributed independently of synthetic augmentation under the same experimental protocol; nevertheless, GAN-generated samples may not fully capture real-world pathological variability, and future studies should validate the framework on large-scale, independent multi-center datasets without relying on synthetic data.

## Conclusion

This study presented an information-theoretic evaluation framework built upon a CNN–LSTM-based model for three-class Alzheimer’s disease classification from structural MRI. The CNN component extracted intra-slice spatial features, while the LSTM component modeled ordered inter-slice spatial dependencies across anatomically arranged MRI slices. The primary contribution of this work is not the introduction of a new network architecture, but the use of post-hoc information-theoretic measures to complement conventional classification metrics.

Using a subject-level evaluation protocol on ADNI data, the CNN–LSTM framework achieved a mean cross-validation accuracy of 96.7% and an F1-score of 92.1%. Renyi mutual information, Renyi divergence, and Henze–Penrose divergence provided complementary evidence regarding information preservation, representation stability, and output-distribution alignment. These measures helped characterize model behavior beyond accuracy alone and may support more transparent comparison of deep learning models in neuroimaging studies.

The results also suggest that GAN-based augmentation can improve performance under the present ADNI-based protocol when synthetic samples are restricted to the training folds. However, the findings should be interpreted with caution because synthetic images may introduce distributional artifacts, and the study was limited to a single structural MRI dataset. External validation on independent multi-center cohorts, direct comparison with 3D CNN and transformer-based volumetric models, and prospective clinical evaluation are required before clinical deployment can be considered. Future work should also investigate whether information-theoretic measures can be incorporated directly into training objectives or regularization strategies.

## Data Availability

This study is based exclusively on secondary analysis of data obtained from the Alzheimer’s Disease Neuroimaging Initiative (ADNI) database (https://adni.loni.usc.edu). Access to ADNI data is provided to qualified researchers following registration and approval of a data use agreement. All data analyzed were fully anonymized and previously collected; no new data were generated for this study.
